# Can next-generation humanized mice that reconstituted with both functional human immune system and hepatocytes model the progression of viral hepatitis to hepatocarcinogenesis?

**DOI:** 10.3389/fmed.2022.1002260

**Published:** 2022-09-23

**Authors:** Jinglong Guo, Siyue Wang, Qi Gao

**Affiliations:** ^1^Department of Cardiovascular Disease, The First Hospital of Jilin University, Changchun, China; ^2^Graduate Program in Immunology and Microbiology, Baylor College of Medicine, One Baylor Plaza, Houston, TX, United States

**Keywords:** humanized mice, humanized immune system, human hepatocytes chimeric, hepatitis virus, liver immunopathogenesis, fibrosis, cirrhosis, hepatocellular carcinoma

## Abstract

Hepatitis B virus (HBV) and Hepatitis C virus (HCV) chronic infections cause liver immunopathological diseases such as hepatitis, fibrosis, cirrhosis, and hepatocellular carcinomas, which are difficult to treat and continue to be major health problems globally. Due to the species-specific hepato-tropism of HBV and HCV, conventional rodent models are limited in their utility for studying the infection and associated liver immunopathogenesis. Humanized mice reconstituted with both functional human immune system and hepatocytes (HIS-HuHEP mice) have been extremely instrumental for *in vivo* studies of HBV or HCV infection and human-specific aspects of the progression of liver immunopathogenesis. However, none of the current HIS-HuHEP mice can model the progression of viral hepatitis to hepatocarcinogenesis which may be a notorious result of HBV or HCV chronic infection in patients, suggesting that they were functionally compromised and that there is still significant space to improve and establish next-generation of HIS-HuHEP mice with more sophisticated functions. In this review, we first summarize the principal requirements to establish HIS-HuHEP mice. We then discuss the respective protocols for current HIS-HuHEP mice and their applications, as well as their advantages and disadvantages. We also raise perspectives for further improving and establishing next-generation HIS-HuHEP mice.

## Introduction

Globally, ~500 million people are chronically infected with Hepatitis B virus (HBV) and Hepatitis C virus (HCV). These chronic infections cause disordered immune responses, progressively resulting in liver immunopathogenesis and diseases such as hepatitis, fibrosis, cirrhosis, and hepatocellular carcinomas (HCC) (1–4). Although the burden of alcohol- and NASH-related HCC is increasing, chronic hepatitis (HBV or HCV) remains a major cause of HCC development worldwide (1–4). Hepatotropic HBV and HCV only infect humans and primates, limiting the availability of animal models for studying virus infection and viral-specific immune responses *in vivo*. Mice transgenic with HBV whole genome, HBV or HCV individual gene, have made significant contributions to our understanding of the hepatitis virus-host interaction *in vivo* (5–7). However, these mice have several major drawbacks, (1) they may not model the entire life cycles of HBV or HCV infection; (2) mouse T cells may be immune tolerant because they consider virus proteins as self-antigens, and consequently, liver immunopathogenesis may not be observed in these mice; (3) most importantly, most findings from mouse studies cannot be applied to humans due to significant incongruences between rodents and humans (5–7). Thus, there has been an urgent and unmet need to generate versatile animal models that not only support highly effective infection of HBV or HCV but also mount viral-specific immune responses (8–10).

Unsurprisingly, such animal models have been attributed to the humanized mice reconstituted with both functional human immune system and hepatocytes (referred as to HIS-HuHEP mice) (8–10). Despite decades of separate efforts to construct and optimize humanized mice with a functional human immune system reconstitution (i.e., HIS mice) and human hepatocytes chimeric mice (i.e., HuHEP mice), the successful establishment of HIS-HuHEP mice was not achieved until recently when several principal requirements were met (8–10). It is worth mentioning that HIS mice are extremely useful for the investigations of human hematopoiesis and immunity, as well as a broad range of human immune diseases *in vivo*, including HIV infection, allo- and xeno-transplantation, autoimmunity, and cancer immunotherapies (11–16). Meanwhile, HuHEP mice showed special values for modeling human hepatotropic pathogen infections including HBV, HCV, and Plasmodium, and testing preventive and therapeutic interventions (8, 17–20). However, neither HIS mice nor HuHEP mice can be used to study the HBV- or HCV-immune interactions and associated liver immunopathology (8, 10). Some kinds of currently reported HIS-HuHEP mice have been shown to develop liver immunopathogenesis including hepatitis and fibrosis/cirrhosis upon HBV or HCV infection, however, neither of them progressed to HCC (8, 10). This is likely because of the limitations of these mouse models.

In this review, we first summarize the principal conditions required for the successful construction of HIS-HuHEP mice. We then discuss the development and applications of currently available HIS-HuHEP mice and their main pros and cons. We also propose future directions to establish functionally sophisticated next generations of HIS-HuHEP mice that could be used to study the progression of viral hepatitis to hepatocarcinogenesis and human-specific therapies.

## Overview of the principal requirements for constructing HIS-HuHEP mice

The generation of humanized mouse models with both a functional human immune system and hepatocytes (either major histocompatibility complex (MHC)-mismatched or autologous) has been successfully achieved recently, although there were last-standing efforts to generate and optimize HIS mice or HuHEP mice, separately. The requirements for constructing such dual humanized mice are far more than those for either HIS mice alone or HuHEP mice alone. To summarize, three primary requirements must be met to construct HIS-HuHEP mice.

### Principal requirement 1: The availability of immunodeficient mouse strains

Highly immunodeficient mice, which do not reject xenogeneic human cells and tissue transplants and support their differentiation and growth, are indispensable for generating humanized mouse models. Nonobese diabetic/severe combined immunodeficiency (NOD/SCID, or genetic nomenclature NOD.Cg-Prkdcscid/J) mice,), and their derivatives NOD.Cg-PrkdcscidIl2rgtm1Sug/JicTac (NOG) and NOD.Cg-PrkdcscidIl2rgtm1Wjl/SzJ (NSG) mice are among the most-commonly used immunodeficient mouse strains today (21–23). NSG and NOG mice show much more defects in T cells, B cells, and natural killer (NK) cells and reduced function of Macrophages and dendritic cells (DCs) than their parental NOD/SCID mice, because they acquire an additional mutation of IL-2 receptor common gamma chain (IL-2rg) that is shared by IL-2, IL-4, IL-7, IL-9, IL-15, and IL-21 cytokines critical for the growth/differentiation/function of T cells, B cells, and NK cells (22–24). In addition, NOD mice lack hemolytic complement and express Sirpa capable of cross-acting with human CD47, which has been reported to significantly reduce the xeno-rejection mediated by the complement-mediated antibody-independent responses and Macrophages/DCs activations because of incompatibility between donor CD47 and recipient Sirpa (i.e., the lack of “don't eat me” signal), respectively, in xenotransplantation settings (25–28). Immunodeficient mice of non-NOD background can also obtain the “don't eat me” signal for Macrophages/DCs to donor human cells through transgenic expression of human Sirpa (29, 30). Other immunodeficient mice with IL-2rg mutation used for human cell/tissue engraftment include Balb/cA-Rag2-/-Il2rg-/- (BRG) and C57BL/6-Rag2-/-Il2rg-/- (B6RG) (24, 31). Even though they, particularly the B6RG, display lower engraftment rates of human cells/tissues than NSG and NOG (24, 31), they might be particularly useful in certain circumstances. They might be easier and faster to acquire additional gene modifications purposely (like below discussed mice with gene modifications to cause mouse liver injury), as well-established mouse genetic techniques rely heavily on their parental strains Balb/c or C57BL/6 mice (32).

### Principal requirement 2: Strategies to provide selective growth advantages for human hepatocytes in mouse liver

To achieve successful repopulation of human hepatocytes in mouse livers, other than the immunodeficiency of the host mice, the signals for selective expansion of transplanted human hepatocytes over resident mouse hepatocytes are remarkably required. To date, there are generally three strategies that have been widely used for providing such signals (33, 34). (1), when newborn mice are used as recipients of human hepatocyte transplantation, the development process of unmatured livers of newborn mice to mature livers of adult mice supports both mouse and human hepatocyte proliferation (33). In this setting, exogenous human hepatocytes, that have fewer growth advantages than resident mouse hepatocytes and compete with them for expansion and colonization niche, usually achieve limited repopulation rates (33). (2), the use of hepatocyte toxic reagents to suppress proliferation or cause cell death of resident mouse hepatocytes. For example, injection of anti-mouse Fas agonist antibody (Jo2) can specifically induce mouse hepatocyte death by recognizing Fas that is preferentially expressed by hepatocytes and initiating Fas-mediated apoptosis pathway (35). Jo2 doesn't recognize human Fas, thus selectively induces the death of host mouse hepatocytes, but not human hepatocytes (36, 37). The doses and periods of Jo2 treatment need to be optimized to cause chronic liver injury, which, instead of fulminant hepatitis, is required to repopulate transplanted human hepatocytes (36, 37). The advantage of Jo2 is to be readily applied to any available immunodeficient mouse strains. (3), the generation of immunodeficient mice carrying genetic modifications to induce mouse hepatocyte death represents the most critical progression in the field, given such mice can achieve high rates of human hepatocyte repopulation that effectively support HBV or HCV infection (8).

Immunodeficient mice harboring gene modifications related to hepatocyte death have been widely reported to achieve high-level repopulation of human hepatocytes upon transplantation. The first such mice are SCID mice expressing uroplasminogen activator (uPA) under the control of albumin promoter (i.e., uPA/CB-17 SCID-bg) (38, 39). Constitutive expression of uPA results in hepatic injury, thereby producing signals for selective expansion of transplanted human hepatocytes. Except for uPA/SCID, there are other uPA-based immunodeficient mouse strains such as uPA/NOG and uPA/Rag2-/- mice. They also showed reasonable levels for repopulating human hepatocytes (40–42). The main disadvantages of above mentioned uPA-based mice include a high rate of neonatal mortality, susceptibility to kidney diseases, low breeding efficiency, small body size and weight, limited time window for transplantation of healthy hepatocytes, and spontaneous transgenic uPA loss due to homologous recombination in resident mouse hepatocytes, that outcompete the repopulation of transplanted human hepatocytes.

The second widely used mouse strain is Rag2-/-IL2rg-/- mice with a knockout of the fumarylacetoacetate hydrolase (Fah) gene (i.e., FRG mice) (43). Fah is a tyrosine catabolic enzyme whose deficiency results in the accumulation of the toxic metabolite fumarylacetoacetate, which causes hepatic injury. The accumulation of fumarylacetoacetate and liver injury can be prevented by administration with 2-(2-nitro-4-trifluoro-methylbenzoyl)-1,3-cyclohexanedione (NTBC), a small molecule that blocks tyrosine catabolism upstream of Fah (44, 45). Withdrawing NTBC administration circularly causes the liver injury of FRG mice, supporting the high-level repopulation of human hepatocytes after transplantation. FRG mice have several advantages over the uPA/SCID strain and its derivatives, including higher rates of human hepatocyte repopulation, ease of breeding, and no spontaneous transgene loss. However, the long-term treatment with NTBC may cause the development of liver cancer and confound the studies of drug metabolism (44, 45).

More recently, two additional such mouse strains have been developed, the TK-NOG and AFC8 mice (46, 47). TK-NOG mice were generated by inducing a liver-specific expression of a herpes simplex virus type 1 thymidine kinase (HSVtk) in NOG mice. The hepatocytes of TK-NOG mice can be selectively deleted *via* administration of ganciclovir (GCV), thus producing signals for selective growth of transplanted human hepatocytes (46). AFC8 mice were derived from immunodeficient mice Balb/cRag2-/-IL2rg-/- (BRG) by expressing a fused FK506 binding protein (FKBP) and caspase 8 under the control of albumin promoter. To induce liver cell death, the AFC8 mice were administered AP20187, a synthetic drug that caused the dimerization of FKBP, resulting in caspase 8-mediated apoptosis of mouse hepatocytes (47).

The uPA transgene-, Fah-/–based, and TK-NOG immunodeficient mice have been shown to repopulate high levels (>70%) of human adult hepatocytes because they can acquire more severe liver injury by indicated gene modifications (8, 39, 43, 46). However, human fetal hepatoblasts that are hepatic precursors capable to differentiate into hepatocytes (48), compared to human adult hepatocytes, were less effective in repopulating mouse livers, as demonstrated in the uPA/NOG mice (42). The reasons for this difference are still largely unknown. The AFC8 mice have only been studied for repopulation of human fetal hepatoblasts, but not for adult hepatocytes yet (47).

### Principal requirement 3: The cell sources of repopulated human immune cells and hepatocytes

#### Human hematopoietic stem/progenitor cells

In currently reported HIS-HuHEP models, human fetal liver-derived CD34+ hematopoietic stem/progenitor cells (HSPCs) were the most commonly used pluripotent stem cells for the reconstitution of multilineage human immune cells, including T cells, B cells, NK cells, Monocytes/macrophages, and DCs (Table 1). Many reports have shown that these cells are superior to HSPCs that are derived from cord blood, bone marrow, or granulocyte-colony stimulating factor (G-CSF) mobilized peripheral blood in terms of the levels of human immune cell reconstitution in mice (49, 50). The latter three types of HSPCs also represent important options for specific research aims, although they have various capabilities of reconstituting human immune cells *in vivo* (49, 50). More importantly, human fetal liver CD34+ HSPCs can be autologous to hepatoblasts derived from the same fetal liver. They develop genetically identical human immune cells and hepatocytes in mice, respectively, upon transplantation. Either neonatal or adult immunodeficient mice can be used as the recipients of HSPCs, albeit the former recipients have been reported to reconstitute human immune cells better (51–53). In addition, the recipient mice will be subjected to sublethal irradiation or busulfan treatment for myeloablation to promote bone marrow engraftment of injected HSPCs.

**Table 1:** List of humanized mice with both functional human immune system and hepatocytes.

**HIS-HuHEP mice (nomenclature)**	**AFC8 hu HSC/Hep mice**	**A2/NSG hu HSC/Hep mice**	**Human-liver-immune mice**	**FRG HIS-Hep mice**	**BRGS-uPA mice**	**uPA-NOG mice**	**Thy/HSC/Hep mice**
Reference	(47)	(36)	(62, 63)	(65)	(55, 56)	(42)	(37)
Immunodeficient strain	BRG	NSG-A2	NSG	FRG	BRGS-uPA	uPA-NOG	NSG
Newborn or adult as recipient	Newborn (HuHEPs+ Hu HSPCs)	Newborn	Newborn	Adult	Newborn and Adult	Adult	Adult
Human hepatocyte (HuHEP) repopulation							
Liver injury induced by	FKBP-Caspase 8 + AP20187	Jo2	N.A.	Fah ko + withdraw NTBC	uPA overexpression	uPA overexpression	Jo2
HuHEPs source	Fetal liver hepatoblasts	Fetal liver hepatoblsts	Fetal liver CD34+ cells	Fetal liver hepatoblasts	Adult hepatocytes	Adult hepatocytes	Fetal liver hepatoblasts
HuHEP repopulation rate	15%	25%	5–10%	up to 50%	20–50%	4.8–6.8%	23%
Human albumin secretion	100 ng/ml	100 ng/ml	26.4 ng/ml	up to 3.3 mg/ml	100–10,000 μg/ml	280 μg/ml	150 ng/ml
Human immune system (HIS) reconstitution							
Myeloablation	Sublethal irradiation	Sublethal irradiation	Sublethal irradiation	Sublethal irradiation	Sublethal irradiation	Treosulfan	Sublethal irradiation
Human HSPCs injected	Fetal liver CD34+ HSPCs	Fetal liver CD34+ HSPCs	Fetal liver CD34+ cells	Fetal liver CD34+ HSPCs	Fetal liver CD34+ HSPCs	Fetal liver CD34+ HSPCs	Fetal liver CD34+ HSPCs
Human CD45+ cells in blood	17%	70%	40%	40%	40%	50–60%	90%
Human T cell developed in	Newborn mouse thymus initially*	Newborn mouse thymus initially*	Newborn mouse thymus initially*	Adult mouse thymus	Adult mouse thymus	Adult mouse thymus	Human fetal thymus
HuHEPs and Hu HSPCs were	Autologous (syngeneic)	Autologous (syngeneic)	Autologous (syngeneic)	Autologous (syngeneic)	Allogeneic (MHC-mismatched)	Allogeneic (MHC-mismatched)	Autologous (syngeneic)
HBV or HCV infection, immune response, and liver disease							
HBV or HCV infection	HCV	HBV	HCV	HBV (or HCV)	HBV	N.A.	N.A.
Viremia	No	Low level	No	High level	High level	N.A.	N.A.
Virus detected in liver	Yes	Yes	Yes	Yes	Yes	N.A.	N.A.
T cell infiltration in liver	Yes	Yes	Yes	No	Yes	N.A.	N.A.
Anti-virus specific antibodies	No	Yes	Yes	No	No	N.A.	N.A.
Anti-virus specific T cells	Yes	Yes	Yes	No	No	N.A.	N.A.
NK activation	Yes	N.A.	N.A.	Yes	Yes	N.A.	N.A.
Monocytes/Macrophage activation	Yes	Yes	Yes	Yes	Yes	N.A.	N.A.
DC activation	Yes	N.A.	N.A.	N.A.	N.A.	N.A.	N.A.
Therapeutic test	N.A.	Anti-HBs neutralizing antibody	Human interferon alpha 2a	N.A.	Nucleoside analog entecavir	N.A.	N.A.
Liver hepatitis, fibrosis/cirrhosis after infection	Yes	Yes	yes	No	No	N.A.	N.A.
Hepatocarcinogenesis	No	No	No	No	No	N.A.	N.A.

N.A., not applicable or not to be determined.

^*^Newborn mouse thymus initially, newborn mice as recipients of HSPC transplantation, thus human T cells developed initially in newborn mouse thymus.

#### Human fetal hepatoblasts and adult hepatocytes

Human fetal hepatoblasts and adult hepatocytes have been most widely used to repopulate the livers in HIS-HuHEP mice (Table 1). Hepatoblasts, which account for 80% of parenchymal cells in fetal liver, are hepatic precursors that can differentiate into hepatocytes and cholangiocytes (48). Even though the exact reasons are unidentified, it has been shown that adult hepatocytes have a greater capacity to expand and repopulate in mouse liver than human fetal hepatoblasts (42, 54). Notably, when transplanted with human fetal liver CD34+ HSPCs and human adult hepatocytes, the developed human immune cells and repopulated human hepatocytes in mice were MHC-mismatched (42, 55, 56). Interestingly, the reconstituted human immune cells didn't mediate allo-rejection of the repopulated MHC-mismatched human hepatocytes in these mice (42, 55, 56), one of the most likely reasons would be that human T cells developed in these mice were functional abnormal (51, 57). One disadvantage of the use of human fetal liver CD34+ HSPCs and human fetal hepatoblasts (and human fetal thymic tissues discussed later) for HIS-HuHEP mice is the need for human fetal tissues. Induced pluripotent stem cells (iPSC) and embryonic stem cells (ESC) have been reported to differentiate into a variety of cell types including functional hepatocyte-like, hematopoietic stem cell-like, and thymus epithelial cells (58–60). They hold great potential to overcome the disadvantage of using human fetal tissues and to be used as valuable cell sources for establishing humanized mice.

Either of the previously mentioned requirements can affect the efficiency and functions of engrafted human immune cells and chimeric human hepatocytes in HIS-HuHEP mice. Reliable and sophisticated models need to comprehensively integrate the requirements. How reliable the HIS-huHEP mice (i.e., human immune cells and human hepatocytes functionally normal and acceptable levels of human cell reconstitution that support their normal functions) are would largely affect their applications, including studying HBV and HCV infection and associated immune responses, hepatitis, liver pathology, and fibrosis/cirrhosis and testing anti-virus regimens and vaccines, and the development of hepatocarcinogenesis from viral hepatitis, which is still the missing link of current HIS-HuHEP mice.

## HIS-HuHEP mice with autologous human immune system and hepatocytes

### AFC8-hu HEP/HSC Mice

Neonatal AFC8 mice that received an intra-liver injection of the mixture of human fetal liver CD34+ HSPCs and autologous hepatoblasts, with the help of AP20187 treatment to induce liver injury, resulted in the reconstitution of multilineages of human immune cells, as well as 15% of the liver repopulated with human hepatocytes (named AFC8-hu HSC/HEP mice) (47, 61). Upon infection by HCV isolates, 50% of the mice displayed a persistent infection, evidenced by the detectable HCV genomic RNA in the livers up to 3 months after infection. The HCV-infected mice mounted elevated immune responses, including HCV-specific T cells, NK cells, plasmacytoid dendritic cells (pDCs), and Macrophages, as well as developed liver immunopathogenesis such as liver injury and fibrosis/cirrhosis, the pathology features commonly observed in patients with chronic HCV infection. Limitations of these models include undetectable HCV viremia in the blood, which is likely attributed to poor human hepatocyte repopulation. Besides, these mice also failed to generate anti-HCV-specific antibodies, suggesting the functions of human B cells might not be fully normal (Table 1).

### A2/NSG-hu HSC/Hep mice

A2/NSG-hu HSC/Hep mice were generated by injection of human fetal liver CD34+ HSPCs and autologous hepatoblasts into the livers of neonatal A2/NSG mice (36) (Table 1). Transgene expression of human leukocyte antigen A2 (HLA-A2), a subset of MHC-class I molecules encoded by A^*^02 alleles, in A2/NSG mice can facilitate the development of human MHC-restricted T cells (57). To promote human hepatocyte repopulation, A2/NSG-hu HSC/Hep mice were treated with Jo2, a mouse-specific anti-Fas agonistic antibody, to induce mouse hepatocyte death. The A2/NSG-hu HSC/Hep mice developed 70% of human CD45+ immune cells in peripheral blood and 25% of human hepatocytes in the livers. Upon HBV infection, ~75% of A2/NSG hu HSC/Hep mice established persistent infection for at least 4 months, evidenced by lasted detectable serum HBV genome and Hepatitis B surface (HBs) antigen, as well as detectable Hepatitis B core (HBc) and HBs antigens in the mouse livers when sacrificed. HBV-infected mice developed virus-specific B cell and T cell responses, evidenced by the production of anti-HBs IgM and anti-HBs IgG antibodies and HB core-specific and Hepatitis B envelope antigen (HB env)-specific T cells, respectively. Moreover, the HBV-infected mice developed chronic hepatitis and fibrosis, important features of liver immunopathogenesis caused by chronic HBV infection in patients. Furthermore, these mice also demonstrated an association between M2-like macrophage activation and immune impairment, and liver fibrosis during chronic HBV infection, similar to clinical scenarios in patients with chronic HBV infection. Finally, treatment with anti-HBs neutralizing antibody (NAb) prevented HBV infection and associated liver immunopathological disease in these mouse models, showing their values in testing anti-HBV therapeutic interventions (36).

### Human-immune-liver (HIL) mice

In human fetal liver, CD34+ cells have been reported to contain three distinct subpopulations: CD34hiCD133hi, CD34loCD133lo, and CD34hiCD133neg. CD34hiCD133hi cells were pluripotent HSPCs, whereas CD34loCD133lo showed potential as hepatic progenitors. When transplanted into NSG mice, they developed multilineages of human immune cells and human hepatocytes, respectively (62). Based on these findings, Keng et al. developed human-immune-liver (HIL) mice by intra-liver injection of general CD34+ cells isolated from the human fetal liver into neonatal NSG mice (63). HIL mice established ~ 40% human immune cells in the blood and 5–10% human hepatocytes in the livers. Of note, these mice didn't undergo liver injury to promote human hepatocyte repopulation. HIL mice supported HCV infection and the HCV-infected mice displayed robust virus-specific immune responses, liver inflammation, and fibrosis. Moreover, human T cells and Macrophages were shown to play critical roles in driving liver inflammation and fibrosis in HCV-infected mice. Finally, HIL mice reproduced the therapeutic effects of a clinically used drug, human interferon alpha 2a, of which treatment inhibited HCV replication and prevented liver disease progression (63). The primary disadvantage of HIL mice, along with the above discussed AFC8 hu HSC/Hep and A2/NSG hu HSC/Hep mice, was the low repopulation rate of human hepatocytes, which likely contributed to low or undetectable viremia upon HBV or HCV infection (Table 1).

### FRG HIS-Hep mice

FRG mice have been reported to allow high-level repopulation of human adult hepatocytes, with the help of cycling withdrawal of NTBC to induce liver damage. More recently, Billerbeck et al. found these mice were less effective to repopulate human fetal hepatoblasts; nonetheless, the repopulation was significantly improved when the mice were treated with human oncostatin M (OSM), an important cytokine for fetal liver development (64). Moving forward, Billerbeck and colleagues performed combined transplantation of human fetal liver CD34+ HSPCs and autologous hepatoblasts into adult FRG mice (65) (Table 1). As expected, FRG HIS-Hep mice not only developed multilineages of human immune cells but also repopulated human hepatocytes with high rates and largely increased the production of albumin (named FRG HIS-Hep mice). Upon HBV or HCV infection, FRG HIS-Hep mice displayed rapid, sustained, and high levels of viremia. The HBV-infected mice showed increased human monocytes and NK cells. However, the HBV-infected mice were not reported to mount T cell and B cell immune responses, nor developed liver immunopathogenesis (65). This suggests that the functions of human immune cells, especially T cells and B cells, in these mice were poor.

## HIS-HuHEP mice with MHC-mismatched human immune cells and human hepatocytes

Using adult NOG-uPA mice as recipients, Gutti et al. compared the repopulation levels of human fetal hepatoblasts and human adult hepatocytes, in the presence of human immune cells differentiated from transplanted human fetal liver CD34+ HSPCs (42). Of note, the human immune cells were autologous to the repopulated human fetal hepatoblasts, whereas they were MHC-mismatched to the engrafted human adult hepatocytes. Surprisingly, human fetal hepatoblasts failed to achieve long-term, high-level repopulation, in contrast, human adult hepatocytes achieved, albeit in the presence of MHC-mismatched human immune cells (42). More recently, by transplantation of human fetal liver CD34+ HSPCs and adult hepatocytes into adult Balb/cRag2-/-IL2rg-/-NOD.sirpa uPAtg/tg mice (BRGS-uPA), Dusseaux et al. reported the generation of the HIS-HUHEP mice, of which human immune cells and hepatocytes were MHC-mismatched as well (55, 56). These two models all demonstrated human adult hepatocytes were not allo-rejected by MHC-mismatched human immune cells, which is likely because human T cells were functionally abnormal. In fact, human T cell function poor has been considered a general problem when they differentiated in the xenogeneic mouse thymus, where HLA-restricted antigen recognition is lacking (51). Transgene expression of HLA-A2 in mice can partially address this issue (36, 57). Notably, these HIS-HUHEP mice didn't develop HBV-specific T cell responses, liver inflammation, and fibrosis, although they supported the HBV infection with a complete life cycle (Table 1). This further highlighted that human T cells developed in these mice were functionally compromised.

## Summary of advantages and disadvantages of above described HIS-HuHEP mice

### HIS-HuHEP mice with functional human immune cells but low rates of human hepatocyte population

Two major disadvantages, low repopulation rates of human hepatocytes and compromised functions of human adaptive immune cells, can divide the HIS-HuHEP mice introduced above into two groups. Group 1 mice included AFC8-hu HEP/HSC, A2/NSG-hu HSC/Hep, and HIL, as they displayed a limited repopulation rate of human hepatocytes (Table 1). Consequently, they were limitedly supportive of hepatitis virus infection. Despite these disadvantages, Group 1 mice, upon HBV or HCV infection, were detected with liver hepatitis and fibrosis. More importantly, they mounted virus-specific B and T cell responses. These represented important advantages that the following mentioned Group 2 mice lacked. Commonly, all the Group 1 mice used newborn immunodeficient mice as recipients of transplanted human cells. Reconstitution of newborn mice, compared to adult mice, has been considered a major improvement, especially for human T cells (52, 53). The thymus of neonatal mice might be less involution than that of adult mice, which could support better human T cell development. It is worth mentioning that human T cell function would likely be comprised even if they developed in the newborn mouse thymus (52, 53).

### HIS-HuHEP mice with high levels of human hepatocyte repopulation but human immune cells functionally compromised

FRG HIS-Hep and the two HIS-HuHEP mice with MHC-mismatched human immune cells and hepatocytes were attributed to Group 2 because they generally didn't develop liver immunopathogenesis and particularly anti-virus specific T cell responses (Table 1). It implied the functions of human T cells in these mice are functionally abnormal, likely because they developed in the thymus of adult recipient mice (51). Nonetheless, the Group 2 mice had a more effective human hepatocyte repopulation that was more supportive of hepatitis virus infection. Of note, the latter two HIS-HuHEP mice of Group 2 (i.e., the ones that were established through transplantation of human fetal liver CD34+ HSPCs and MHC-mismatched adult hepatocytes) used adult human hepatocytes for mouse liver repopulation. Although FRG HIS-Hep mice used human fetal hepatoblasts instead of adult hepatocytes, they received the treatment of human OSM, a cytokine that can promote fetal hepatoblast maturation (64, 65). In this regard, adult human hepatocytes appeared to repopulate mouse livers more efficiently than human fetal hepatoblasts, representing a shared point across Group 2 mice. Each discussed mouse model had its disadvantages and advantages. Interchanges of the advantages would provide promising ideas to improve the HIS-HuHEP mouse models, i.e., improved levels of human hepatocyte repopulation that supports effective hepatitis virus infection and improved functions of the reconstituted human immune system simultaneously.

### The reconstitution of humanized immune microenvironment locally in the liver of current HIS-HuHEP mice is compromised

The liver immune microenvironment encompasses parenchymal cells (mostly hepatocytes), immune cells, and stromal cells including liver sinusoidal endothelial cells (LSECs), fibroblasts, and hepatic stellate cells, all of which are extensively cross-regulated to maintain liver hemostasis and induce pathological responses in diseases (66). Except for hepatocytes and immune cells, liver stromal cells also play important roles in the development of liver immunopathogenesis caused by chronic HBV or HCV infection (66). LSECs are unique liver-resident antigen presenting cells (APCs) that can induce CD8 T cell responses to HBV infection (67). Mouse LSECs might be less effective than their human counterparts in responding to human immune mediators upon HBV or HCV infection in humanized mice. Notably, the reconstitution of a humanized liver immune microenvironment containing human stromal cells in humanized mice remains a significant challenge nowadays. It also represents one of the major drawbacks that could cause compromised local T cell responses to HBV or HCV in current HIS-HuHEP mice.

### The progression of viral hepatitis to hepatocarcinogenesis is still the missing link in current HIS-HuHEP mice

Hepatocellular carcinoma (HCC) is increasing in incidence and is one of the major causes of cancer-related death. Cirrhosis due to chronic viral hepatitis (HBV and HCV) remains the leading cause of the disease worldwide (1–4). Liver cirrhosis causes the accumulation of progressive gene mutations that can lead to cancer (68, 69). HBV chronic infection also plays direct oncogenic effects by expressing oncogenic proteins, insertional mutagenesis, and causing chromosomal instability (69, 70). Whether HCV infection plays a direct oncogenic role in HCC development remains debated (1–4). Humanized mice that can model the human-specific development of HCC from chronic HBV or HCV infection will be highly valuable. Although HIS-HuHEP mice raise the significant potential for this, all the current mice were not overserved the hepatocarcinogenesis. For HIS-HuHEP mice of above Group 1, it could be explained by the low-rate repopulation of human hepatocytes and low or ineffective HBV or HCV infection. The functional compromise of human immune cells (especially T cells) and the failure of developing liver immunopathogenesis could lead to their inability to progress to HCC in Group 2 HIS-HuHEP mice. Of note, neither of current HBV- or HCV- infected HIS-HuHEP mice has been subjected to any well-known comorbidities related to HCC risk, which might be one of the important reasons why they didn't develop HCC. How cooperate between liver immunopathogenesis caused by chronic HBV or HCV infection and the comorbidities to drive hepatocellular carcinogenesis in patients remains largely unknown (71). Functionally sophisticated HIS-HuHEP mice can be used as an instrumental *in vivo* platform to answer this question. It is worth mentioning that improved human immune system in HIS-HuHEP mice would develop acute responses that eliminate the virus and control liver immunopathogenesis upon HBV or HCV infection. High HBV load has been reported to result in less efficient immune control and hepatitis in humanized mice (56). It suggests that chronic infection of HBV or HCV in humanized mice could be achieved through increasing the viral inoculum.

## Optimize the current accomplishments to make next-generation HIS-HuHEP mice

**Figure 1:**
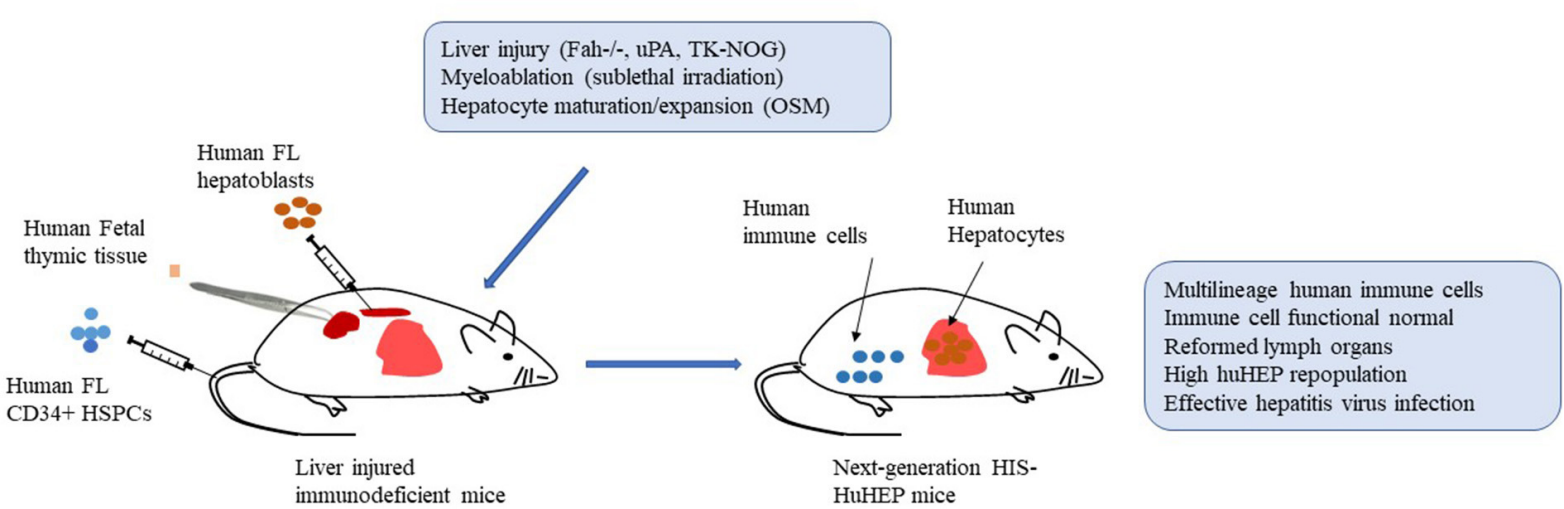
Establishment of next-generation humanized mice with both functional human immune systems and autologous hepatocytes (HIS-HuHEP mice). Autologous human fetal liver CD34+ hematopoietic stem/progenitor cells (HSPCs; intravenously), fetal hepatoblasts (intrasplenic), and fetal thymic tissues (under kidney capsule) are transplanted into immunodeficient mice with gene modifications of liver injury, such as Fah-/-Rag2-/-IL2rg-/- (FRG mice), SCID mice expressing uroplasminogen activator (uPA) in liver (uPA/SCID mice), and NOG mice expressing a herpes simplex virus type 1 thymidine kinase (HSVtk) in liver (TK-NOG mice). Sublethal irradiation is used to promote bone marrow engraftment of HSPCs. Liver injury caused by indicated gene modification and human Oncostatin M (OSM) treatment improve the liver repopulation with human fetal hepatoblasts. More importantly, human T cells are developing in human thymic tissue. Overall, the resultant HIS-HuHEP mice display improved reconstitution levels and functions of human immune cells, the formation of lymph organs such as spleen and lymph nodes, high-level repopulation of human hepatocytes, and highly effective infection with Hepatitis B virus (HBV) or Hepatitis C virus (HCV).

Accordingly, further improvement and optimization need to be placed on the functions of human adaptive and innate immune cells and repopulation rates of human hepatocytes in next-generation HIS-HuHEP mice simultaneously. Next-generation HIS-HuHEP mice are expected to display high-level repopulation rates of human immune cells as well as hepatocytes with improved functions. Moreover, they reform histological structures of lymph organs such as spleen and lymph nodes and establish a local liver immune microenvironment composed of human hepatocytes, multilineages of human immune cells, and human stromal cells. Such mice support highly effective HBV or HCV infection and mount functional robust immune responses against the infections. By increasing the viral inoculum, HIS-HuHEP mice could establish a chronic HBV or HCV infection that results in advanced liver immunopathogenesis including hepatitis, fibrosis/cirrhosis (Figure 1).

### Improving human T cell functions in HIS-HuHEP mice

Human T cells developing in the xenogeneic mouse thymus have been increasingly documented to be functionally poor, presumably because of the absence of HLA-restricted antigen recognition (51, 57). Even though they express human HLA-A2, human immune system (HIS) mice that used the newborn immunodeficient mice as recipients of human HSPCs only achieved a limited improvement of human T cell functions (51, 57). To further solve this issue, two groups developed a new generation of HIS mice through combined transplantation of human fetal thymic tissue (under kidney capsule) and fetal liver CD34+ HSPCs (i.v.) in 2006 (12, 72). The resultant Thy/HSC (or termed BLT elsewhere) mice reconstituted high levels of multilineage human immune cells such as T cells, B cells, Monocytes/Macrophages, and DCs in blood and lymphoid organs (12, 72). The engrafted human fetal thymic tissue was determined to form a typical histology structure and support potent thymopoiesis. More importantly, the Thy/HSC mice reformed secondary lymph organs and mounted specific adaptive and innate immune responses *in vivo* in multiple disease settings (12, 72). Of note, human T cells in Thy/HSC mice have been widely reported to be functionally improved, evidenced by the rejection of various allo-grafts (14, 73). Therefore, the introduction of human fetal thymus into current HIS-HuHEP mice would represent a promising strategy to improve human T cell functions (12, 74–76).

HIS-HuHEP mice with autologous human immune systems and hepatocytes might be more reliable than those with MHC-mismatched human immune cells and hepatocytes, especially when human T cells are expected to be functionally normal. More recently, we determined the feasibility of generating humanized mice with combined transplantation of autologous human fetal thymic tissue (under kidney capsule), CD34+ HSPCs (i.v.), and fetal hepatoblasts (intrasplenic), with the help of Jo2 treatment to promote the hepatoblast repopulation (referred to as Thy/HSC/Hep mice) (37). Other than the similar levels of human immune cell reconstitution in the blood and lymph organs to conventional Thy/HSC mice, Thy/HSC/Hep mice acquired human hepatocyte repopulation and improved engraftment of human Macrophages, NK cells, and DCs in the livers. The repopulation rate of human hepatocytes in these mice was ~23% on average, a similar level to that in the above discussed Group 1 mice, suggesting the use of human fetal hepatoblasts to repopulate mouse livers conferred a common drawback of low repopulation rates. Notably, even though human T cells that developed in human thymic tissue in Thy/HSC/Hep mice were expected to be functionally normal, how they would perform in physiological settings like HBV or HCV infections hasn't been reported yet (37).

### To improve engraftment and function of myeloid and NK cells in HIS-HuHEP mice

The reconstitution and function of human myeloid and NK cells were usually compromised in humanized mice, due to the lack of sufficient crosstalk of human cells with mouse cytokines (GM-CSF, M-CSF, IL-3, IL-15, Flt-3l, etc.) (77, 78). Introducing these cytokines can remarkedly improve the development and function of human innate immune cells in mice (77, 78). However, the constitutive existence of these cytokines might cause vigorous proinflammatory responses and widespread tissue inflammation, which would likely confound the evaluation of interested immune functions in humanized mice (79). Interestingly, the repopulated human hepatocytes have been reported to contribute to the improved engraftment of human Macrophages, NK cells, and DCs in the livers of the humanized mice by producing cytokines and chemokines important for immune cell development, differentiation, tissue migration and retention, such as IL-3, IL-15, GM-CSF, M-CSF, MCP-1, CXCL-1, and CXCL-10 (37, 80). This indicates that even though there were no additional transgenes in HIS-HuHEP animals, the repopulated human hepatocytes could still produce the cytokines and chemokines necessary to enhance the engraftment and activities of myeloid cells and NK cells.

### Improving the repopulation levels of human fetal hepatoblasts in HIS-HuHEP mice

In fact, human adult hepatocytes have been demonstrated to be the superior cells for repopulating mouse livers, which supported a completed life cycle of HBV or HCV infection (8). However, their source is limited (34, 81), and they are not easy to be matched with autologous HSPCs and autologous thymic tissues, which are cell sources for immune cells and support functional T cell development, respectively. In this regard, human fetal hepatoblasts would remain especially valuable. Given their limited ability to repopulate mouse livers, how to improve the repopulation of human fetal hepatoblasts would be one of the essential steps to establish next-generation HIS-HuHEP mice. The generation of FRG HIS-Hep mice has provided a good example (65). Firstly, FRG mice, one of the best immunodeficient strains with gene modification to cause liver injury, were used as recipients. Secondly, FRG mice were treated with human OSM to promote the maturation and repopulation of transplanted human fetal hepatoblasts. More importantly, the repopulated human fetal hepatoblasts (likely matured hepatocytes after growing and human OSM treatment *in vivo*) in FRG HIS-Hep mice supported a full life cycle of HBV or HCV infection (65). Therefore, the use of gene modification-induced liver injury mice such as FRG, as well as uPA-based and TK-NOG immunodeficient mice and the methods (including treatment with human OSM) that can promote maturation and/or expansion of human fetal hepatoblasts would provide promising options to improve the repopulation of human fetal hepatoblasts in HIS-HuHEP mice.

### HIS mice transplanted with a bioengineered human liver

As discussed above, the establishment of a reliable liver immune microenvironment containing human stromal cells (especially LSECs) in HIS-HuHEP mice remains challenging. The generation of bioengineered human liver tissues that contain multiple types of human cell components and mimic liver microstructure has been widely reported with the advancement and integration of Biomaterial engineering, ES- or iPS cell reprogramming, tissue three-dimensional (3D) bioprinting, and 3D organoid culture technologies (82). The HIS mice transplanted with a bioengineered human liver (HIS-huLiver mice) would hold the remarkable potential to reconstruct an improved liver local immune microenvironment that could mount more effective immune responses upon HBV or HCV infection. However, whether human immune cells effectively penetrate transplanted bioengineered human liver tissue in HIS-huLiver mice either before or after HBV or HCV infection has a significant impact on the studies.

## Concluding remarks

Chronic HBV or HCV infection and its associated liver immunopathogenesis are well-known as major risk factors for the development of hepatocellular carcinomas (HCC), one of the deadliest cancers globally (1–4, 68, 70). Humanized mice reconstituted with both functional human immune system and human hepatocytes (HIS-HuHEP mice) have been highly valuable in the study of human-specific liver immune responses, inflammation, pathology, and fibrosis/cirrhosis caused by the infections of hepatotropic viruses HBV and HCV. Additionally, they have been utilized to test clinically used vaccinations and therapeutics. However, neither current hepatitis virus-infected HIS-HuHEP mice have been reported to develop this disease, most likely due to their major disadvantages discussed above (i.e., compromised function of the human immune system and low rate of human hepatocyte repopulation) and the lack of comorbidities related to HCC development in patients. Further improvement and optimization are urgently required to establish next-generation HIS-HuHEP mice, which are expected to carry both improved human immune system and human hepatocytes in terms of both functions and repopulation rates. By increasing the viral inoculum and the use of comorbidities, such HIS-HuHEP mice would develop a chronic infection that features a full cycle of HBV or HCV infection, high-level viremia, impaired immune responses, and advanced hepatitis and fibrosis/cirrhosis, which causes the accumulation of progressive mutations leading to hepatocarcinogenesis. Besides, the HBV or HCV that establishes chronic infection in repopulated human hepatocytes in HIS-HuHEP mice could also accelerate hepatocarcinogenesis through playing their direct oncogenic effects.

## Author contributions

All authors contributed to conceptualization, writing, editing, and approved the submitted version.

## Conflict of interest

The authors declare that the research was conducted in the absence of any commercial or financial relationships that could be construed as a potential conflict of interest. The handling Editor ZH declared a shared affiliation with the authors QG and JG at the time of review.

## Publisher's note

All claims expressed in this article are solely those of the authors and do not necessarily represent those of their affiliated organizations, or those of the publisher, the editors and the reviewers. Any product that may be evaluated in this article, or claim that may be made by its manufacturer, is not guaranteed or endorsed by the publisher.
